# 3-Methyl­anilinium hydrogen phthalate

**DOI:** 10.1107/S1600536811054353

**Published:** 2011-12-23

**Authors:** Ming-Liang Liu

**Affiliations:** a College of Chemistry and Chemical Engineering, Southeast University, Nanjing 211189, People’s Republic of China

## Abstract

The asymmetric unit of the title salt, C_7_H_10_N^+^·C_8_H_5_O_4_^−^, consists of two 3-methyl­phenyl­ammonium cations and two hydrogen phthalate anions. There are strong intra­molecular O—H⋯O hydrogen bonds in the virtually planar (r.m.s. deviations = 0.054 Å) phthalate anions. In the crystal, the cations and anions are connected *via* an extensive sytem of N—H⋯O hydrogen bonds into a corrugated layer extended parallel to (001).

## Related literature

The title compound was investigated as part of work looking for new ferroelectric compounds. For background to ferroelectric compounds consisting of organic cations and inorganic anions, see: Fu *et al.* (2011 ▶); Ye *et al.* (2010 ▶). For a related structure, see: Kadirvelraj *et al.* (1996 ▶).
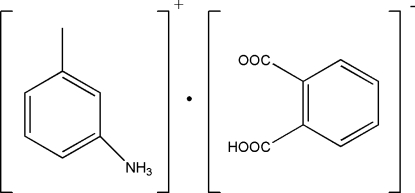


## Experimental

### 

#### Crystal data

C_7_H_10_N^+^·C_8_H_5_O_4_^−^
*M* *_r_* = 273.28
Monoclinic, 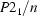

*a* = 7.9325 (16) Å
*b* = 17.931 (4) Å
*c* = 19.575 (4) Å
β = 93.37 (3)°
*V* = 2779.5 (10) Å^3^
*Z* = 8
Mo *K*α radiation
μ = 0.10 mm^−1^
*T* = 293 K
0.36 × 0.32 × 0.28 mm

#### Data collection

Rigaku Mercury2 diffractometer
Absorption correction: multi-scan (*CrystalClear*; Rigaku, 2005 ▶) *T*_min_ = 0.963, *T*_max_ = 0.971
22962 measured reflections
4906 independent reflections
2489 reflections with *I* > 2σ(*I*)
*R*_int_ = 0.090

#### Refinement

*R*[*F* ^2^ > 2σ(*F* ^2^)] = 0.066
*wR*(*F* ^2^) = 0.165
*S* = 1.03
4906 reflections
368 parameters
H-atom parameters constrained
Δρ_max_ = 0.18 e Å^−3^
Δρ_min_ = −0.21 e Å^−3^

### 

Data collection: *CrystalClear* (Rigaku, 2005 ▶); cell refinement: *CrystalClear*; data reduction: *CrystalClear*; program(s) used to solve structure: *SHELXS97* (Sheldrick, 2008 ▶); program(s) used to refine structure: *SHELXL97* (Sheldrick, 2008 ▶); molecular graphics: *SHELXTL* (Sheldrick, 2008 ▶); software used to prepare material for publication: *SHELXTL*.

## Supplementary Material

Crystal structure: contains datablock(s) I, global. DOI: 10.1107/S1600536811054353/gk2431sup1.cif

Structure factors: contains datablock(s) I. DOI: 10.1107/S1600536811054353/gk2431Isup2.hkl

Supplementary material file. DOI: 10.1107/S1600536811054353/gk2431Isup3.mol

Supplementary material file. DOI: 10.1107/S1600536811054353/gk2431Isup4.cml

Additional supplementary materials:  crystallographic information; 3D view; checkCIF report

## Figures and Tables

**Table 1:** Hydrogen-bond geometry (Å, °)

*D*—H⋯*A*	*D*—H	H⋯*A*	*D*⋯*A*	*D*—H⋯*A*
N2—H2*A*⋯O5^i^	0.89	1.87	2.739 (3)	166
N2—H2*B*⋯O2^ii^	0.89	1.93	2.815 (3)	178
N2—H2*C*⋯O8	0.89	1.90	2.789 (3)	178
N1—H1*A*⋯O6^i^	0.89	1.94	2.826 (3)	177
N1—H1*B*⋯O1^i^	0.89	1.91	2.784 (3)	166
N1—H1*C*⋯O4^iii^	0.89	1.90	2.788 (3)	172
O3—H3⋯O2	0.82	1.58	2.392 (3)	173
O7—H7⋯O6	0.82	1.57	2.392 (3)	180

Symmetry codes: (i) 
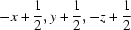
; (ii) 
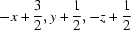
; (iii) 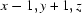.

**Fig. 1.**
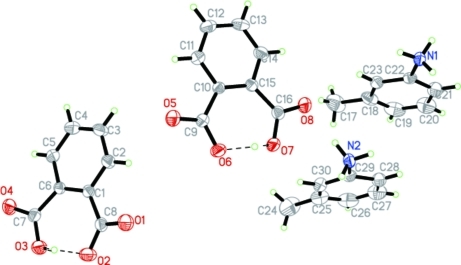
The structure of the title compound, showing the atomic numbering scheme with 30% probability displacement ellipsoids.

**Fig. 2.**
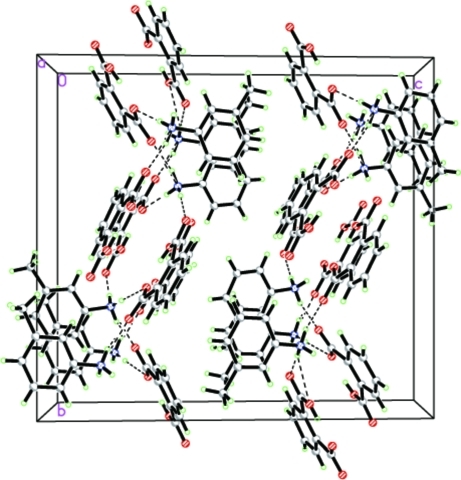
Crystal structure of the title compound with view along the a axis. Dashed lines indicate hydrogen bonds.

## supplementary crystallographic information

### Comment

Recently much attention has been devoted to simple molecular-ionic compounds containing inorganic ions and organic ions owing to the tunability of their special structural features and their potential ferroelectric properties (Fu *et al.*, 2011; Ye *et al.*,2010;).

In our laboratory, the title compound has been synthesized and its crystal structure is herein reported. The title salt, C_7_H_10_N^+^.C_8_H_5_O_4_^–^ has an asymmetric unit that consists of two 3-methylphenylamonium cations and two phthalate anions (Fig 1). In the crystal structure, there are some O—H—O intramolecular hydrogen bonds in the phthalate anions, the phthalate anion is almostly planar. The 3-methylphenylamonium cations and phthalate anions are associated by N—H···O hydrogen-bonding interaction (Fig. 2, Table 1).

The dielectric constant of the compound as a function of temperature indicates that the permittivity is basically temperature-independent (*ε* = C/(T–T_0_)), suggesting that this compound is not ferroelectric or there may be no distinct phase transition occurring within the measured temperature range (below the melting point).

### Experimental

3.21 g (0.03 mol) of 3-methylaniline was dissolved in 30 ml ethanol to which 4.98 g (0.03 mol) of phthalic acid was added to afford the solution without any precipitation under stirring at the ambient temperature. Single crystals suitable for X-ray structure analysis were obtained by slow evaporation of the solution after 3 days.

### Refinement

H atoms were placed in calculated positions (N—H = 0.89 Å; O—H = 0.82 Å; C—H = 0.93 Å for C*sp^2^* atoms and C—H = 0.96 Å for C*sp^3^* atoms) with *U*_iso_ values *U*_iso_ = 1.2*U*eq(C*sp^2^*,O) and *U*_iso_ = 1.5*U*eq(C*sp^3^*,N) and allowed to ride.

### Crystal data

**Table:**

C_7_H_10_N^+^·C_8_H_5_O_4_^−^	*F*(000) = 1152
*M**_r_* = 273.28	*D*_x_ = 1.306 Mg m^−^^3^
Monoclinic, *P*2_1_/*n*	Melting point: 413 K
Hall symbol: -P 2yn	Mo *K*α radiation, λ = 0.71073 Å
*a* = 7.9325 (16) Å	Cell parameters from 4906 reflections
*b* = 17.931 (4) Å	θ = 3.4–25.0°
*c* = 19.575 (4) Å	µ = 0.10 mm^−^^1^
β = 93.37 (3)°	*T* = 293 K
*V* = 2779.5 (10) Å^3^	Block, colourless
*Z* = 8	0.36 × 0.32 × 0.28 mm

### Data collection

**Table:**

Rigaku Mercury2 diffractometer	4906 independent reflections
Radiation source: fine-focus sealed tube	2489 reflections with *I* > 2σ(*I*)
graphite	*R*_int_ = 0.090
Detector resolution: 13.6612 pixels mm^-1^	θ_max_ = 25.0°, θ_min_ = 3.1°
ω scans	*h* = −9→9
Absorption correction: multi-scan (*CrystalClear*; Rigaku, 2005)	*k* = −21→21
*T*_min_ = 0.963, *T*_max_ = 0.971	*l* = −23→23
22962 measured reflections	

### Refinement

**Table:**

Refinement on *F*^2^	Secondary atom site location: difference Fourier map
Least-squares matrix: full	Hydrogen site location: inferred from neighbouring sites
*R*[*F*^2^ > 2σ(*F*^2^)] = 0.066	H-atom parameters constrained
*wR*(*F*^2^) = 0.165	*w* = 1/[σ^2^(*F*_o_^2^) + (0.0672*P*)^2^] where *P* = (*F*_o_^2^ + 2*F*_c_^2^)/3
*S* = 1.03	(Δ/σ)_max_ = 0.003
4906 reflections	Δρ_max_ = 0.18 e Å^−^^3^
368 parameters	Δρ_min_ = −0.21 e Å^−^^3^
0 restraints	Extinction correction: *SHELXL97* (Sheldrick, 2008), Fc^*^=kFc[1+0.001xFc^2^λ^3^/sin(2θ)]^-1/4^
Primary atom site location: structure-invariant direct methods	Extinction coefficient: 0.0038 (9)

### Special details

**Table:**

Geometry. All esds (except the esd in the dihedral angle between two l.s. planes) are estimated using the full covariance matrix. The cell esds are taken into account individually in the estimation of esds in distances, angles and torsion angles; correlations between esds in cell parameters are only used when they are defined by crystal symmetry. An approximate (isotropic) treatment of cell esds is used for estimating esds involving l.s. planes.
Refinement. Refinement of F^2^ against ALL reflections. The weighted R-factor wR and goodness of fit S are based on F^2^, conventional R-factors R are based on F, with F set to zero for negative F^2^. The threshold expression of F^2^ > 2sigma(F^2^) is used only for calculating R-factors(gt) etc. and is not relevant to the choice of reflections for refinement. R-factors based on F^2^ are statistically about twice as large as those based on F, and R- factors based on ALL data will be even larger.

### Fractional atomic coordinates and isotropic or equivalent isotropic displacement parameters (Å^2^)

**Table:**

	*x*	*y*	*z*	*U*_iso_*/*U*_eq_	
N2	0.4142 (3)	0.70027 (13)	0.16189 (12)	0.0493 (7)	
H2A	0.3797	0.7446	0.1761	0.074*	
H2B	0.4874	0.6809	0.1932	0.074*	
H2C	0.3258	0.6700	0.1556	0.074*	
N1	−0.0859 (3)	0.85234 (13)	0.15519 (12)	0.0471 (7)	
H1A	−0.0165	0.8742	0.1865	0.071*	
H1B	−0.1163	0.8078	0.1704	0.071*	
H1C	−0.1773	0.8805	0.1473	0.071*	
O2	0.8571 (3)	0.14134 (13)	0.23666 (12)	0.0671 (7)	
O6	0.3588 (3)	0.41675 (12)	0.24495 (12)	0.0680 (7)	
O3	0.8400 (3)	0.02633 (13)	0.17473 (13)	0.0627 (7)	
H3	0.8455	0.0637	0.1989	0.094*	
O4	0.6480 (3)	−0.04790 (12)	0.13023 (12)	0.0701 (7)	
O1	0.6943 (3)	0.22617 (13)	0.27712 (13)	0.0703 (7)	
O5	0.2074 (3)	0.32453 (13)	0.27620 (13)	0.0780 (8)	
O7	0.3337 (3)	0.53387 (12)	0.18671 (13)	0.0626 (7)	
H7	0.3424	0.4936	0.2065	0.094*	
O8	0.1401 (3)	0.60431 (12)	0.13895 (12)	0.0676 (7)	
C10	0.0608 (3)	0.41746 (16)	0.21047 (14)	0.0378 (7)	
C1	0.5594 (4)	0.13815 (16)	0.20140 (14)	0.0399 (8)	
C15	0.0443 (4)	0.48752 (16)	0.17754 (14)	0.0402 (7)	
C6	0.5481 (4)	0.06991 (16)	0.16526 (15)	0.0401 (7)	
C22	0.0006 (4)	0.84311 (18)	0.09148 (15)	0.0443 (8)	
C9	0.2169 (4)	0.38404 (18)	0.24608 (16)	0.0459 (8)	
C11	−0.0827 (4)	0.37315 (18)	0.21255 (16)	0.0522 (9)	
H11	−0.0725	0.3266	0.2333	0.063*	
C2	0.4145 (4)	0.18156 (18)	0.20308 (16)	0.0514 (9)	
H2	0.4205	0.2266	0.2266	0.062*	
C7	0.6849 (4)	0.01263 (19)	0.15662 (16)	0.0480 (8)	
C29	0.4959 (4)	0.70911 (18)	0.09736 (16)	0.0469 (8)	
C8	0.7121 (4)	0.17078 (18)	0.24167 (16)	0.0461 (8)	
C23	0.0200 (4)	0.77319 (18)	0.06485 (17)	0.0517 (9)	
H23	−0.0179	0.7314	0.0876	0.062*	
C3	0.2629 (4)	0.1606 (2)	0.17137 (18)	0.0626 (10)	
H3A	0.1686	0.1912	0.1732	0.075*	
C12	−0.2372 (4)	0.3946 (2)	0.18566 (18)	0.0617 (10)	
H12	−0.3299	0.3631	0.1877	0.074*	
C30	0.5465 (4)	0.64590 (19)	0.06421 (17)	0.0558 (9)	
H30	0.5298	0.5994	0.0838	0.067*	
C28	0.5197 (4)	0.77878 (19)	0.0716 (2)	0.0651 (10)	
H28	0.4882	0.8211	0.0951	0.078*	
C25	0.6213 (5)	0.6503 (3)	0.0026 (2)	0.0732 (11)	
C14	−0.1142 (4)	0.50854 (19)	0.15125 (16)	0.0567 (9)	
H14	−0.1270	0.5547	0.1300	0.068*	
C13	−0.2539 (4)	0.4634 (2)	0.15549 (18)	0.0662 (10)	
H13	−0.3591	0.4796	0.1379	0.079*	
C5	0.3929 (4)	0.04985 (18)	0.13383 (17)	0.0555 (9)	
H5	0.3842	0.0051	0.1099	0.067*	
C21	0.0560 (4)	0.9060 (2)	0.06061 (19)	0.0663 (10)	
H21	0.0413	0.9527	0.0800	0.080*	
C19	0.1539 (5)	0.8296 (3)	−0.02783 (19)	0.0838 (13)	
H19	0.2068	0.8255	−0.0688	0.101*	
C18	0.0977 (4)	0.7657 (2)	0.0029 (2)	0.0682 (11)	
C4	0.2521 (4)	0.0941 (2)	0.13703 (19)	0.0672 (11)	
H4	0.1497	0.0790	0.1159	0.081*	
C20	0.1342 (5)	0.8988 (3)	0.0001 (2)	0.0880 (13)	
H20	0.1736	0.9408	−0.0218	0.106*	
C27	0.5930 (5)	0.7842 (3)	0.0088 (3)	0.0940 (15)	
H27	0.6089	0.8306	−0.0109	0.113*	
C26	0.6419 (5)	0.7202 (3)	−0.0240 (2)	0.0900 (14)	
H26	0.6907	0.7248	−0.0659	0.108*	
C24	0.6775 (6)	0.5823 (3)	−0.0342 (2)	0.1177 (18)	
H24A	0.5976	0.5428	−0.0290	0.176*	
H24B	0.7864	0.5670	−0.0152	0.176*	
H24C	0.6846	0.5936	−0.0819	0.176*	
C16	0.1788 (4)	0.54521 (18)	0.16718 (16)	0.0465 (8)	
C17	0.1206 (5)	0.6898 (3)	−0.0274 (2)	0.1080 (16)	
H17A	0.0371	0.6821	−0.0642	0.162*	
H17B	0.1080	0.6525	0.0072	0.162*	
H17C	0.2313	0.6861	−0.0444	0.162*	

### Atomic displacement parameters (Å^2^)

**Table:**

	*U*^11^	*U*^22^	*U*^33^	*U*^12^	*U*^13^	*U*^23^
N2	0.0534 (17)	0.0370 (15)	0.0571 (17)	0.0049 (13)	−0.0004 (14)	0.0010 (13)
N1	0.0494 (16)	0.0363 (15)	0.0553 (17)	−0.0015 (13)	−0.0004 (13)	0.0001 (13)
O2	0.0426 (14)	0.0666 (16)	0.0910 (18)	0.0052 (13)	−0.0060 (13)	−0.0292 (14)
O6	0.0423 (14)	0.0593 (15)	0.101 (2)	−0.0084 (12)	−0.0082 (13)	0.0324 (14)
O3	0.0503 (15)	0.0542 (16)	0.0834 (19)	0.0053 (12)	0.0017 (13)	−0.0211 (13)
O4	0.0704 (17)	0.0445 (15)	0.0935 (19)	0.0018 (13)	−0.0101 (14)	−0.0154 (14)
O1	0.0647 (16)	0.0536 (15)	0.0921 (19)	0.0035 (13)	−0.0002 (13)	−0.0288 (14)
O5	0.0611 (17)	0.0530 (16)	0.119 (2)	−0.0025 (13)	−0.0001 (15)	0.0312 (16)
O7	0.0471 (15)	0.0452 (14)	0.095 (2)	−0.0076 (12)	−0.0034 (13)	0.0204 (13)
O8	0.0647 (17)	0.0446 (14)	0.0925 (19)	−0.0009 (12)	−0.0043 (13)	0.0181 (13)
C10	0.0354 (18)	0.0393 (18)	0.0392 (17)	−0.0020 (15)	0.0062 (14)	−0.0048 (14)
C1	0.0398 (19)	0.0389 (18)	0.0416 (18)	0.0002 (15)	0.0091 (15)	0.0057 (15)
C15	0.0421 (19)	0.0412 (19)	0.0380 (17)	0.0019 (16)	0.0075 (14)	−0.0056 (15)
C6	0.0393 (19)	0.0383 (18)	0.0429 (18)	0.0005 (15)	0.0036 (15)	0.0084 (15)
C22	0.0355 (18)	0.053 (2)	0.0440 (19)	0.0000 (16)	−0.0028 (15)	0.0059 (17)
C9	0.044 (2)	0.042 (2)	0.052 (2)	−0.0034 (17)	0.0049 (16)	0.0038 (16)
C11	0.048 (2)	0.052 (2)	0.057 (2)	−0.0032 (18)	0.0058 (17)	0.0013 (17)
C2	0.048 (2)	0.049 (2)	0.058 (2)	0.0047 (18)	0.0083 (17)	0.0028 (17)
C7	0.049 (2)	0.045 (2)	0.050 (2)	−0.0040 (18)	0.0025 (16)	0.0032 (17)
C29	0.0395 (19)	0.047 (2)	0.053 (2)	−0.0051 (16)	−0.0031 (16)	0.0107 (17)
C8	0.049 (2)	0.0370 (19)	0.052 (2)	0.0021 (17)	0.0058 (17)	−0.0005 (16)
C23	0.046 (2)	0.050 (2)	0.058 (2)	−0.0005 (17)	−0.0047 (17)	−0.0075 (18)
C3	0.040 (2)	0.069 (3)	0.080 (3)	0.010 (2)	0.0075 (18)	0.011 (2)
C12	0.041 (2)	0.072 (3)	0.072 (3)	−0.011 (2)	0.0047 (18)	−0.003 (2)
C30	0.059 (2)	0.051 (2)	0.058 (2)	−0.0056 (19)	0.0037 (18)	0.0004 (19)
C28	0.056 (2)	0.051 (2)	0.088 (3)	−0.0011 (19)	−0.001 (2)	0.023 (2)
C25	0.065 (3)	0.102 (3)	0.053 (2)	−0.008 (2)	0.006 (2)	−0.007 (3)
C14	0.047 (2)	0.060 (2)	0.062 (2)	0.0070 (19)	−0.0002 (18)	0.0053 (18)
C13	0.036 (2)	0.087 (3)	0.074 (3)	0.009 (2)	−0.0050 (18)	0.000 (2)
C5	0.048 (2)	0.048 (2)	0.069 (2)	−0.0042 (18)	−0.0068 (18)	0.0001 (18)
C21	0.065 (3)	0.060 (2)	0.075 (3)	0.000 (2)	0.009 (2)	0.019 (2)
C19	0.065 (3)	0.139 (4)	0.048 (2)	0.017 (3)	0.013 (2)	0.016 (3)
C18	0.053 (2)	0.095 (3)	0.055 (2)	0.018 (2)	−0.008 (2)	−0.020 (2)
C4	0.047 (2)	0.070 (3)	0.083 (3)	−0.003 (2)	−0.009 (2)	0.005 (2)
C20	0.081 (3)	0.105 (4)	0.080 (3)	0.006 (3)	0.018 (3)	0.035 (3)
C27	0.070 (3)	0.097 (4)	0.115 (4)	−0.007 (3)	0.006 (3)	0.065 (3)
C26	0.064 (3)	0.137 (4)	0.069 (3)	0.000 (3)	0.003 (2)	0.028 (3)
C24	0.109 (4)	0.149 (4)	0.099 (3)	−0.023 (3)	0.041 (3)	−0.058 (3)
C16	0.049 (2)	0.044 (2)	0.046 (2)	0.0031 (18)	0.0020 (17)	−0.0047 (16)
C17	0.103 (4)	0.128 (4)	0.092 (3)	0.027 (3)	−0.002 (3)	−0.054 (3)

### Geometric parameters (Å, °)

**Table:**

N2—C29	1.462 (4)	C29—C30	1.377 (4)
N2—H2A	0.8900	C23—C18	1.399 (5)
N2—H2B	0.8900	C23—H23	0.9300
N2—H2C	0.8900	C3—C4	1.368 (4)
N1—C22	1.468 (4)	C3—H3A	0.9300
N1—H1A	0.8900	C12—C13	1.371 (5)
N1—H1B	0.8900	C12—H12	0.9300
N1—H1C	0.8900	C30—C25	1.377 (5)
O2—C8	1.275 (3)	C30—H30	0.9300
O6—C9	1.271 (3)	C28—C27	1.394 (5)
O3—C7	1.284 (3)	C28—H28	0.9300
O3—H3	0.8200	C25—C26	1.372 (5)
O4—C7	1.230 (3)	C25—C24	1.496 (5)
O1—C8	1.225 (3)	C14—C13	1.379 (4)
O5—C9	1.223 (3)	C14—H14	0.9300
O7—C16	1.281 (3)	C13—H13	0.9300
O7—H7	0.8200	C5—C4	1.375 (4)
O8—C16	1.226 (3)	C5—H5	0.9300
C10—C11	1.390 (4)	C21—C20	1.375 (5)
C10—C15	1.414 (4)	C21—H21	0.9300
C10—C9	1.509 (4)	C19—C20	1.367 (5)
C1—C2	1.390 (4)	C19—C18	1.381 (5)
C1—C6	1.413 (4)	C19—H19	0.9300
C1—C8	1.522 (4)	C18—C17	1.500 (5)
C15—C14	1.382 (4)	C4—H4	0.9300
C15—C16	1.508 (4)	C20—H20	0.9300
C6—C5	1.391 (4)	C27—C26	1.381 (6)
C6—C7	1.511 (4)	C27—H27	0.9300
C22—C21	1.364 (4)	C26—H26	0.9300
C22—C23	1.370 (4)	C24—H24A	0.9600
C11—C12	1.361 (4)	C24—H24B	0.9600
C11—H11	0.9300	C24—H24C	0.9600
C2—C3	1.373 (4)	C17—H17A	0.9600
C2—H2	0.9300	C17—H17B	0.9600
C29—C28	1.364 (4)	C17—H17C	0.9600
			
C29—N2—H2A	109.5	C13—C12—H12	120.5
C29—N2—H2B	109.5	C29—C30—C25	121.2 (3)
H2A—N2—H2B	109.5	C29—C30—H30	119.4
C29—N2—H2C	109.5	C25—C30—H30	119.4
H2A—N2—H2C	109.5	C29—C28—C27	117.6 (4)
H2B—N2—H2C	109.5	C29—C28—H28	121.2
C22—N1—H1A	109.5	C27—C28—H28	121.2
C22—N1—H1B	109.5	C26—C25—C30	116.9 (4)
H1A—N1—H1B	109.5	C26—C25—C24	121.0 (4)
C22—N1—H1C	109.5	C30—C25—C24	122.1 (4)
H1A—N1—H1C	109.5	C13—C14—C15	122.3 (3)
H1B—N1—H1C	109.5	C13—C14—H14	118.8
C7—O3—H3	109.5	C15—C14—H14	118.8
C16—O7—H7	109.5	C12—C13—C14	119.7 (3)
C11—C10—C15	117.8 (3)	C12—C13—H13	120.1
C11—C10—C9	114.2 (3)	C14—C13—H13	120.1
C15—C10—C9	127.9 (3)	C4—C5—C6	122.1 (3)
C2—C1—C6	117.9 (3)	C4—C5—H5	119.0
C2—C1—C8	114.0 (3)	C6—C5—H5	119.0
C6—C1—C8	128.1 (3)	C22—C21—C20	118.5 (4)
C14—C15—C10	118.0 (3)	C22—C21—H21	120.7
C14—C15—C16	113.4 (3)	C20—C21—H21	120.7
C10—C15—C16	128.5 (3)	C20—C19—C18	122.1 (4)
C5—C6—C1	118.3 (3)	C20—C19—H19	119.0
C5—C6—C7	113.4 (3)	C18—C19—H19	119.0
C1—C6—C7	128.3 (3)	C19—C18—C23	117.9 (4)
C21—C22—C23	122.8 (3)	C19—C18—C17	122.1 (4)
C21—C22—N1	117.5 (3)	C23—C18—C17	120.0 (4)
C23—C22—N1	119.7 (3)	C3—C4—C5	119.8 (3)
O5—C9—O6	119.4 (3)	C3—C4—H4	120.1
O5—C9—C10	119.8 (3)	C5—C4—H4	120.1
O6—C9—C10	120.8 (3)	C19—C20—C21	119.8 (4)
C12—C11—C10	123.2 (3)	C19—C20—H20	120.1
C12—C11—H11	118.4	C21—C20—H20	120.1
C10—C11—H11	118.4	C26—C27—C28	119.7 (4)
C3—C2—C1	122.6 (3)	C26—C27—H27	120.1
C3—C2—H2	118.7	C28—C27—H27	120.1
C1—C2—H2	118.7	C25—C26—C27	122.6 (4)
O4—C7—O3	119.0 (3)	C25—C26—H26	118.7
O4—C7—C6	119.5 (3)	C27—C26—H26	118.7
O3—C7—C6	121.4 (3)	C25—C24—H24A	109.5
C28—C29—C30	121.9 (3)	C25—C24—H24B	109.5
C28—C29—N2	119.8 (3)	H24A—C24—H24B	109.5
C30—C29—N2	118.3 (3)	C25—C24—H24C	109.5
O1—C8—O2	120.9 (3)	H24A—C24—H24C	109.5
O1—C8—C1	119.4 (3)	H24B—C24—H24C	109.5
O2—C8—C1	119.7 (3)	O8—C16—O7	118.8 (3)
C22—C23—C18	118.9 (3)	O8—C16—C15	119.5 (3)
C22—C23—H23	120.6	O7—C16—C15	121.6 (3)
C18—C23—H23	120.6	C18—C17—H17A	109.5
C4—C3—C2	119.3 (3)	C18—C17—H17B	109.5
C4—C3—H3A	120.4	H17A—C17—H17B	109.5
C2—C3—H3A	120.4	C18—C17—H17C	109.5
C11—C12—C13	118.9 (3)	H17A—C17—H17C	109.5
C11—C12—H12	120.5	H17B—C17—H17C	109.5

### Hydrogen-bond geometry (Å, °)

**Table:**

*D*—H···*A*	*D*—H	H···*A*	*D*···*A*	*D*—H···*A*
N2—H2A···O5^i^	0.89	1.87	2.739 (3)	166
N2—H2B···O2^ii^	0.89	1.93	2.815 (3)	178
N2—H2C···O8	0.89	1.90	2.789 (3)	178
N1—H1A···O6^i^	0.89	1.94	2.826 (3)	177
N1—H1B···O1^i^	0.89	1.91	2.784 (3)	166
N1—H1C···O4^iii^	0.89	1.90	2.788 (3)	172
O3—H3···O2	0.82	1.58	2.392 (3)	173
O7—H7···O6	0.82	1.57	2.392 (3)	180

Symmetry codes: (i) −*x*+1/2, *y*+1/2, −*z*+1/2; (ii) −*x*+3/2, *y*+1/2, −*z*+1/2; (iii) *x*−1, *y*+1, *z*.
